# Potential Role of Amino Acids in the Adaptation of Chicks and Market-Age Broilers to Heat Stress

**DOI:** 10.3389/fvets.2020.610541

**Published:** 2021-01-08

**Authors:** Vishwajit S. Chowdhury, Guofeng Han, Hatem M. Eltahan, Shogo Haraguchi, Elizabeth R. Gilbert, Mark A. Cline, John F. Cockrem, Takashi Bungo, Mitsuhiro Furuse

**Affiliations:** ^1^Laboratory of Stress Physiology and Metabolism, Faculty of Arts and Science, Kyushu University, Fukuoka, Japan; ^2^Laboratory of Regulation in Metabolism and Behavior, Graduate School of Bioresource and Bioenvironmental Science, Kyushu University, Fukuoka, Japan; ^3^Department of Biochemistry, Showa University School of Medicine, Tokyo, Japan; ^4^School of Neuroscience, Virginia Polytechnic Institute and State University, Blacksburg, VA, United States; ^5^School of Veterinary Science, Massey University, Palmerston North, New Zealand; ^6^Department of Bioresource Science, Graduate School of Biosphere Science, Hiroshima University, Higashi-Hiroshima, Japan

**Keywords:** amino acids metabolism, body temperature, heat stress, growth performance, poultry

## Abstract

Increased average air temperatures and more frequent and prolonged periods of high ambient temperature (HT) associated with global warming will increasingly affect worldwide poultry production. It is thus important to understand how HT impacts poultry physiology and to identify novel approaches to facilitate improved adaptation and thereby maximize poultry growth, health and welfare. Amino acids play a role in many physiological functions, including stress responses, and their relative demand and metabolism are altered tissue-specifically during exposure to HT. For instance, HT decreases plasma citrulline (Cit) in chicks and leucine (Leu) in the embryonic brain and liver. The physiological significance of these changes in amino acids may involve protection of the body from heat stress. Thus, numerous studies have focused on evaluating the effects of dietary administration of amino acids. It was found that oral l-Cit lowered body temperature and increased thermotolerance in layer chicks. When l-Leu was injected into fertile broiler eggs to examine the cause of reduction of Leu in embryos exposed to HT, *in ovo* feeding of l-Leu improved thermotolerance in broiler chicks. *In ovo* injection of l-Leu was also found to inhibit weight loss in market-age broilers exposed to chronic HT, giving rise to the possibility of developing a novel biotechnology aimed at minimizing the economic losses to poultry producers during summer heat stress. These findings and the significance of amino acid metabolism in chicks and market-age broilers under HT are summarized and discussed in this review.

## Introduction

The 0.9°C rise in the world's average surface temperature since the late 19th century has been mainly caused by the increase in carbon dioxide and other human-made emissions into the atmosphere (1). This warming has mostly happened in the past 35 years, with the five warmest years on record having been since 2010 (1). The year 2020 was no exception, with a very high ambient temperature (HT) worldwide in the summer. The HT is a growing challenge for all living organisms, including chickens. In general, because they do not possess sweat glands, but instead rely on evaporative cooling (panting) as main thermoregulatory mechanism, HT is stressful to chickens and interferes with their ability to maintain a homeothermic body temperature (2). The exposure of chickens to HT can cause hyperthermia (3–5). If heat production exceeds heat dissipation capacity, HT can induce stress in chickens (6). This heat stress decreases food intake and body weight (BW) gain, ultimately hindering production and increasing mortality (7–9). Balnave and Oliva (10) reported that absorption of arginine (Arg), an essential amino acid in chickens, significantly decreased when chickens are exposed to heat stress. Several reports showed that the dietary supplementation of certain essential amino acids can mitigate the problems resulting from heat stress in birds (11–16). Increased dietary levels of certain amino acids could be useful to counteract the negative effects of heat stress in chickens. Thus, it is crucial to better understand the importance of this dietary strategy, as well as the role of the amino acids in the metabolism of heat-stressed birds.

Traditionally, it is thought that chicks are not subjected to heat stress. However, series of studies showed that chicks around 2 weeks old are susceptible to HT (35–40°C) when compared with the control thermoneutral temperature (30°C; 4, 5). Several free amino acids have been found to increase in the blood, brain, and muscle of layer chicks within a short time (15 or 30 min) following exposure to HT at 35°C (17); however, when layer chicks were exposed to prolonged HT at 35°C for 48 h, most of these amino acids decreased in the brain and plasma (18). On the basis of these findings, we noticed that citrulline (Cit), which increased in the plasma of layer chicks after short-term heat stress (15 or 30 min at 35°C) and decreased as a result of long-term heat stress (24 or 48 h at 35°C), acts hypothermically in chicks when administered to them orally (19, 20). In addition, thermal manipulation (TM) during embryogenesis was associated with reduced concentrations of leucine (Leu) in the brain and liver of broiler embryos. Notably, *in ovo* injection of l-Leu afforded thermotolerance in broiler chicks (21, 22). Thus, those amino acids that either increase or decrease in chicks, depending on the length of exposure to heat stress, may be used as biomarkers of heat stress.

In this review, we summarize how heat stress affects amino acid metabolism, with a particular focus on the means by which l-Cit and l-Leu afford thermotolerance. Furthermore, we describe the use of *in ovo* feeding of l-Leu to attenuate the BW reduction that occurs in market-age broilers under heat stress.

## Metabolism of Amino Acids in Heat-Exposed Embryos and Chicks

Animal proteins are commonly consisted of 20 amino acids. In growth-phase chicks, 11 of these [arg, histidine, isoleucine, Leu, lysine (Lys), methionine, phenylalanine, threonine, tryptophan, valine, and glycine] are essential amino acids, while the remaining are non-essential (23). Heat stress induces catabolism in organisms (24) in order to provide energy to counter the heat stress. Therefore, any variations in free amino acids that occur under heat stress (17, 18, 25, 26) can be assumed to be the result of catabolic activity. All tissue contains enzymes for amino acid catabolism and synthesis, but their expression and activity varies depending on the tissue's metabolic requirements. In catabolism, deamination/deamidation reactions occur in amino acids, which are followed either by reamination of the carbon skeleton that results to produce non-essential amino acids or by channeling the carbon skeleton into the Krebs cycle. Then, it is either oxidized, funneled toward gluconeogenesis via pyruvate carboxylase, or converted from pyruvate into acetate for the synthesis of fatty acid (27). Glycine is an integral part of the uric acid molecule, and whenever a molecule of uric acid is excreted, a molecule of glycine lost also occurred, particularly during the catabolic phase (28). Amino acids are well-documented to function not only as protein constituents but also as important physiological and behavioral regulators, and this includes the regulation of stress responses (29–34). While there have not been many investigations into the altered free amino acid concentrations found in chickens exposed to heat stress, Ito et al. (17) observed increases in several free essential and non-essential amino acids in the plasma, muscle, and brain in layer chicks following short-term heat stress (15–30 min at 35°C); however, they also found significant reductions in the levels of some other amino acids.

It is still unknown why various amino acids increase in the plasma under short-term heat stress. It is interesting that during short-term heat stress (35°C, 15 or 30 min; 17) the free amino acids have been found to be highly concentrated, in complete contrast to the situation during long-term heat stress (35°C, 24 or 48 h; 18), which was characterized by a reduction in most of the free amino acids. For instance, there was a reduction in tryptophan, Cit, and ornithine (Orn) in the plasma of layer chicks exposed to long-term heat stress (18), whereas the same amino acids increased during short-term heat stress (17). Levels of a variety of free amino acids in the chick brain and breast muscle are also affected by heat stress (17). Interestingly, however, the free amino acids found in the brain and skeletal muscle differ for the most part from those in plasma (17). This suggests that changes in free amino acid concentrations may vary by tissue and be related to tissue-specific enzymatic activity in connection with amino acid metabolism and protein turnover. The concentration of 3-methyl histidine, a marker of proteolysis (35, 36), was found to decline in response to short-term heat stress (17), which suggests that there is a reduction in protein degradation under short-term heat stress (35°C, 15 or 30 min; 17). There is always a balance in the body between protein synthesis and protein degradation, so under short-term heat stress protein synthesis may also be decreased, it may lead to increase in the free amino acid pool in the tissue.

TM during embryogenesis involves increasing the incubation temperature, leading to neonatal chicks (37) and chickens (38) acquiring thermotolerance under HT. We recently reported a significant reduction in some amino acids, including Leu, Lys, and phenylalanine, in the brain and liver of embryos subjected to TM (21). To summarize, amino acid metabolic activity can be influenced by heat stress in broiler embryos and layer chicks.

## Amino Acids Afford Thermotolerance in Heat-Exposed Chicks

When l-Cit, l-Orn, and l-Arg were administered in the left ventricle of the brain, there was no reduction in body temperature (19); however, orally administered l-Cit, but not l-Arg or l-Orn, did decrease it under control thermoneutral temperature (19). Furthermore, oral l-Cit lowered the body temperature in layer chicks under heat exposure (20). These results indicate that l-Cit has a hypothermic role.

Nitric oxide (NO) is produced when l-Arg is converted to l-Cit by NO synthase (39) and may act as a hypothermic factor in chicks because thermoregulation has been proposed to be an important physiological function of NO (40), occurring through cutaneous dissipation (41). However, our findings suggest that NO may not be the main factor in l-Cit–dependent reduction of body temperature and thermotolerance (20). Further research is needed to find out the factor(s) involved in l-Cit–dependent reduction of body temperature. It could be concluded that the production of NO may not be a significant factor in l-Cit–dependent reduction of body temperature. As orally administered l-Cit affords thermotolerance in layer chicks, this amino acid could possibly be proposed as a novel nutritional candidate in assisting poultry to cope with heat stress.

A further finding was that there was a significant reduction in Leu in the embryonic brain and liver as a result of the TM. *In ovo* feeding of l-Leu led to hypothermia in broiler chicks at hatching (21). Metabolic activity was also high during embryogenesis after *in ovo* feeding of l-Leu. Lipid metabolism increased in broiler embryos and chicks as a result of *in ovo* administration of l-Leu, which might have been due to greater mitochondrial activity, as Liang et al. (42) reported that l-Leu and its metabolites [α-ketoisocaproate and β-hydroxy-β-methylbutyrate] can stimulate mitochondrial biogenesis and oxidative activities. Plasma triacylglycerol (TG), non-esterified fatty acids (NEFAs), and ketone bodies were higher in broiler chicks fed l-Leu *in ovo* under heat stress than in heat-exposed control chicks. One might surmise that broiler chicks would benefit from *in ovo* feeding of l-Leu because fat generates less heat, and dietary fats have well-known beneficial effects in hot-weather feeding programs (8).

**Figure 1:**
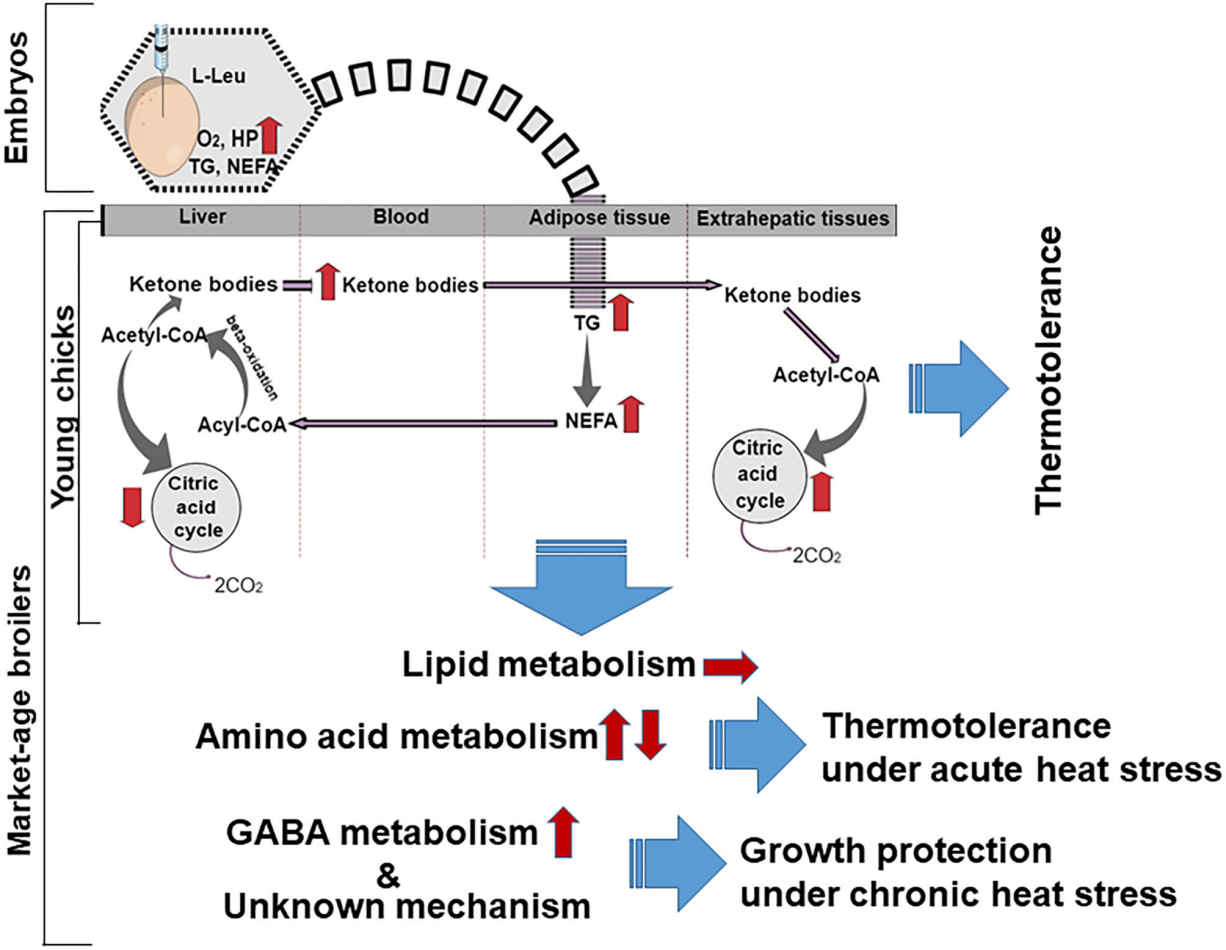
Schematic overview of metabolic possibilities resulting from *in ovo* administration of l-Leu in embryos, young broiler chicks, and market-age broilers. The broken curved line indicates a possible lipid metabolic memory extending from the embryo to the young chick. Arrows indicate the progression (→), increase (↑), or reduction (↓) of metabolites and metabolic processes. l-Leu, l-leucine; TG, triacylglycerol; NEFA, non-esterified fatty acid; HP, heat production; O_2_, oxygen; CO_2_, carbon dioxide; GABA, γ-aminobutyric acid.

As high levels of plasma ketone bodies were found in broiler chicks subjected to l-Leu administration *in ovo*, the liver might be assumed to produce and release more ketone bodies into the bloodstream (22). As a result, a higher demand for acetyl-CoA may occur in the liver, ultimately stimulating the β-oxidation of fatty acids for the production of more acetyl-CoA. Fatty acid oxidation is heavily exergonic in comparison with glucose oxidation, with a high ATP yield (43). Yahav (44) surmised that hyperthermia during heat stress in meat-producing broilers might be the result of a reduced investment in energy under such conditions. Therefore, the fact that the energy produced through lipid metabolism in male broiler chicks given l-Leu is more readily available might assist in affording thermotolerance under heat stress (Figure 1). However, we have not observed any changes in lipid metabolism in chronic heat–exposed market-age broilers subjected to *in ovo* l-Leu feeding (45; Figure 1). Thus, it might be predicted that lipid metabolic activity, which was greater in embryos administered l-Leu *in ovo*, could persist until the neonatal period.

## *IN OVO* Administration of l-Leu Affords Thermotolerance, Supports Growth, and Modulates Amino Acid Metabolism in Market-Age Broilers

As stated above, *in ovo* administration of l-Leu affords thermotolerance in broiler chicks. However, it is important practically to examine whether this effect continues until market age. Han et al. (45) conducted a study where broilers (4 or 6 weeks old) injected with l-Leu *in ovo* were examined under acute and chronic heat stress conditions. They measured changes in body temperature and BW, as well as amino acid metabolism and plasma metabolites. After *in ovo* administration of l-Leu, male broilers (29 or 30 days old) were subjected to acute heat stress (30 ± 1°C for 120 min) or to chronic heat stress (over 30 ± 1°C; aged from 15 to 44 days old). Under acute heat stress, the increased body temperature was found to be suppressed in broilers receiving the *in ovo* administration of l-Leu, without affecting food intake, plasma TG, NEFA, ketone bodies, glucose, lactic acid, or thyroid hormones. Under chronic heat stress, *in ovo* administration of l-Leu increased the daily body temperature. Of note, under chronic heat stress, *in ovo* administration of l-Leu resulted in a higher daily BW gain in comparison with the control group. Furthermore, under acute heat stress, there was a significant increase in several essential amino acids, including Leu and isoleucine, in the liver, and a decrease in their concentration in the plasma, following *in ovo* administration of l-Leu. These findings indicated that *in ovo* administration of l-Leu in broilers up to market age produces thermotolerance under conditions of acute heat stress chiefly by altering the amino acid metabolism.

Although there is limited information available on the *in ovo* injection of amino acids on the performance of broilers under heat stress, dietary administration of some amino acids was found to be beneficial related to growth and meat quality under heat stress. For instance, El-Naggar et al. (46) reported that dietary supplementation of γ-aminobutyric acid (GABA) increased food intake and BW gain in broilers under heat stress. It was found that dietary glutamine improved the antioxidative state of broiler muscles under heat stress (47). Del Vesco et al. (48) showed that dietary methionine supplementation improved protein deposition in acute heat-exposed broilers. Therefore, amino acids could play important role to protect the growth of broilers under heat stress.

In birds, body temperature is considered to be a reliable indicator of thermotolerance acquisition (44). Previously, we have demonstrated that *in ovo* administration of l-Leu in broilers under acute heat stress produced thermotolerance by lowering body temperature and reducing the mRNA expression of heat-shock proteins (22, 49). In market-age broilers, enhanced body temperature due to acute heat stress was lowered by l-Leu, suggesting that *in ovo* administration of l-Leu affords thermotolerance under acute, but not chronic, heat stress until market age is reached. It could be that the reduced body temperature resulted from less heat production, from greater heat loss, or from both. Neither food intake nor plasma metabolites (TG, NEFA, and ketone bodies) were influenced by l-Leu administration following 120 min of heat exposure in market-age broilers, suggesting that food intake and lipid metabolism may not contribute much to l-Leu–induced thermotolerance (45). Thyroid hormones, in particular triiodothyronine (T_**3**_), are important in modifying the metabolic rate, which influences body-temperature regulation (44). *In ovo* administration of l-Leu (34.5 μmol) had no effect on T_3_ concentrations, even when there was a reduction in body temperature during hatching (50). Thus, as suggested by Han et al. (45), plasma thyroid hormones are not influenced by l-Leu under conditions of acute heat stress. TM during embryogenesis, which affords thermotolerance, results in a reduction in thyroid hormones at hatching and in posthatch chicks, as well as in chicks exposed to heat stress (51). l-Leu–mediated thermotolerance could be assumed to be a different mechanism to that of TM. Interestingly, *in ovo* administration of l-Leu in broilers resulted in an increase in body temperature under chronic heat stress. Under continuous heat stress, higher body temperature might be considered to be an adaptive strategy in the broilers treated with l-Leu, because the increased body temperature has been found to enhance sensible heat loss (44). However, the mechanisms underlying the various responses of body temperature under short-term and chronic heat stress are not clear, and as mentioned by Han et al. (45), molecular and physiological analyses are needed.

Chronic heat stress in broilers has negative effects on feed intake, BW gain, and feed-conversion ratios (52). However, *in ovo* administration of l-Leu has been found to attenuate the reduction in BW induced by heat stress in broilers (45). In conditions of continuous heat stress, between 31 and 44 days of age, the difference in BW between the *in ovo* water–administered control and the l-Leu–administered groups tends to be reduced. It is well-known that long-term feed restriction during heat waves in the summer leads to an improvement in heat resistance and thermotolerance, although significantly reducing broiler performance (53). It could be thought that there is a conflict between feed restriction and thermotolerance because feed restriction causes nutritional deficiency in broilers. However, the l-Leu–treated group showed a significantly higher BW compared with the control heat-exposed group, where at 23 days of age the average BW of the l-Leu–administered group was 8.4% higher than that of the control group (45). *In ovo* administration of l-Leu has been reported to enhance growth in chicken embryos (54), and in neonatal pigs, muscle protein synthesis has been found to be improved by dietary l-Leu supplementation (55, 56). Because *in ovo* administration of l-Leu appears to maintain a normal growth under cyclic heat stress (45), it might be assumed that *in ovo* administration of l-Leu accelerates protein synthesis to maintain growth in broilers under heat stress.

Acute heat stress induces catabolic activity in organisms (24). It is well-documented that the liver plays a vital role in the regulation of metabolism, including amino acid metabolism, and it regulates many physiological processes affected by heat stress (57). Many essential amino acids, including Leu, Lys, and isoleucine, were found to be significantly increased in the liver and decreased in the plasma following *in ovo* administration of l-Leu in comparison with control broilers. Acute heat stress alters amino acid metabolism in chicks (17) and decreases some free amino acid concentrations in the brain and plasma of chick embryos (49). During heat stress, higher amino acid concentrations in the liver might be expected, in order to match other tissues' energy need. Han et al. (45) reported higher hepatic concentrations and lower plasma concentrations of amino acids in the l-Leu–treated group than in the control group, which suggests that there were stronger heat-stress responses related to amino acid metabolism in broilers treated with l-Leu. Thus, *in ovo* administration of l-Leu might improve amino acid metabolism during acute heat stress. Amino acids are derived from catabolized proteins and provide carbon backbones for glucose or fatty acid production (57); thus, l-Leu administration might enhance energy metabolism in broilers, and high energy is needed to maintain thermoregulation under heat stress (44). Han et al. (45) showed that the concentration of Lys in the liver increased without any reduction in food intake after 120 min of heat stress, which might indicate that this is a form of metabolic support for various organs via the blood circulatory system, because it has been shown that the dietary Lys requirement increases during heat stress (58). In broilers, food intake was not affected, and body temperature was lowered significantly following l-Leu treatment under 120-min heat stress (45). With 180-min heat stress, food intake was reduced significantly, and Lys concentrations remained higher in the plasma and brain in l-Leu–treated broiler chicks in comparison with control chicks (49). Limesand et al. (59) reported that l-Lys oxidation was significantly increased under chronic hypoglycemia for supporting the growth of fetus. The higher concentrations of Lys in the broiler liver fed l-Leu *in ovo* (43) may be connected to increasing β-oxidation for a higher need of energy supply under heat stress. This suggests that, under heat stress, increased Lys might be a source of higher energy for organs, including the brain, because Lys supplementation compensated for heat stress–induced feed-intake suppression (60). Methionine has been reported to be involved in the expression of stress-related genes, and it provides cellular protection against oxidative stress (61). It has also been reported that supplementation of branched-chain amino acids (Leu, isoleucine, and valine) accelerated protein synthesis and assisted in recovery following a heat-related injury (62). Thus, the changes in amino acid metabolism related to *in ovo* administration of l-Leu could contribute to thermotolerance in broilers, rather than to lipid metabolism as previously predicted in broiler chicks (Figure 1; 22). Future studies are expected to clarify the mechanisms involved in greater detail. The diencephalon is the area of the brain concerned with thermoregulation (44), and oxidative damage has been found here under conditions of prolonged heat stress (18). Han et al. (45) reported a significantly higher concentration of GABA in the brain of the broilers fed l-Leu *in ovo* in comparison with the controls under conditions of chronic heat stress. Al Wakeel et al. (63) reported that supplementation with GABA countered the adverse effects of chronic heat stress on growth, antioxidant status, and immune function in broilers. It might be predicted that increased diencephalic GABA concentration from *in ovo* administration of l-Leu would reduce the negative effects caused by heat stress because GABA is a major inhibitory neurotransmitter, which plays an important role in controlling excitability (64), and it might therefore have helped to protect growth under conditions of chronic heat stress (Figure 1).

## Conclusion

Heat stress causes changes in amino acid metabolism in chicks and market-age broilers. Research collectively points to l-Cit and l-Leu because the levels of these two amino acids were affected by heat stress, and treatment with them afforded thermotolerance in chicks and market-age broilers, respectively. Notably, *in ovo* administration of l-Leu supported a higher BW in market-age broilers in comparison with the control group under conditions of heat stress. Heat stress presents a major problem for poultry production both today and in the future, and our study and others (53, 65–69) can contribute to alleviating this serious global challenge.

## Author Contributions

All authors listed have made a substantial, direct and intellectual contribution to the work, and approved it for publication.

## Conflict of Interest

The authors declare that the research was conducted in the absence of any commercial or financial relationships that could be construed as a potential conflict of interest.
